# Protective Antioxidant and Antiapoptotic Effects of ZnCl_2_ in Rat Pancreatic Islets Cultured in Low and High Glucose Concentrations

**DOI:** 10.1371/journal.pone.0046831

**Published:** 2012-10-03

**Authors:** Jessica Duprez, Leticia P. Roma, Anne-Françoise Close, Jean-Christophe Jonas

**Affiliations:** Université Catholique de Louvain, Institut de Recherche Expérimentale et Clinique, Pôle d’Endocrinologie, Diabète et Nutrition, Brussels, Belgium; Universidad Miguel Hernández de Elche, Spain

## Abstract

**Aim/Hypothesis:**

Rat pancreatic islet cell apoptosis is minimal after prolonged culture in 10 mmol/l glucose (G10), largely increased in 5 mmol/l glucose (G5) and moderately increased in 30 mmol/l glucose (G30). This glucose-dependent asymmetric V-shaped profile is preceded by parallel changes in the mRNA levels of oxidative stress-response genes like *Metallothionein 1a* (*Mt1a*). In this study, we tested the effect of ZnCl_2_, a potent inducer of *Mt1a*, on apoptosis, mitochondrial oxidative stress and alterations of glucose-induced insulin secretion (GSIS) induced by prolonged exposure to low and high *vs.* intermediate glucose concentrations.

**Methods:**

Male Wistar rat islets were cultured in RPMI medium. Islet gene mRNA levels were measured by RTq-PCR. Apoptosis was quantified by measuring islet cytosolic histone-associated DNA fragments and the percentage of TUNEL-positive β-cells. Mitochondrial thiol oxidation was measured in rat islet cell clusters expressing “redox sensitive GFP” targeted to the mitochondria (mt-roGFP1). Insulin secretion was measured by RIA.

**Results:**

As observed for *Mt1a* mRNA levels, β-cell apoptosis and loss of GSIS, culture in either G5 or G30 *vs.* G10 significantly increased mt-roGFP1 oxidation. While TPEN decreased *Mt1a*/*2a* mRNA induction by G5, addition of 50–100 µM ZnCl_2_ to the culture medium strongly increased *Mt1a/2a* mRNA and protein levels, reduced early mt-roGFP oxidation and significantly decreased late β-cell apoptosis after prolonged culture in G5 or G30 *vs.* G10. It did not, however, prevent the loss of GSIS under these culture conditions.

**Conclusion:**

ZnCl_2_ reduces mitochondrial oxidative stress and improves rat β-cell survival during culture in the presence of low and high *vs.* intermediate glucose concentrations without improving their acute GSIS.

## Introduction

Type 2 diabetes results from the combination of insulin resistance and defective glucose stimulation of insulin secretion by the endocrine pancreas. The latter defect is due to a reduction in pancreatic β-cell mass and function [1], [2] that has been diversely attributed to low grade inflammation, mitochondrial oxidative stress or endoplasmic reticulum stress [3]. In this context, we and others have previously shown that, after prolonged culture in the presence of a large range of glucose concentrations, rat islet cell apoptosis follows an asymmetric V-shaped profile with a minimum in 10 mmol/l, a large increase in 5 mmol/l and a moderate increase in 30 mmol/l glucose [4], [5]. These changes were preceded by parallel changes in the mRNA levels of oxidative stress-response genes such as metallothionein 1a (*Mt1a*), heme oxygenase 1 (*Hmox1*) and *c-Myc*, suggesting a possible link between early β-cell oxidative stress and their subsequent apoptosis during prolonged culture in either low or high *vs.* intermediate glucose concentrations [4]. To date, despite considerable debate about whether glucose reduces or increases β-cell oxidative stress [6]–[11], a V-shaped glucose-response curve for β-cell oxidative stress has not been reported yet.

**Figure 1:**
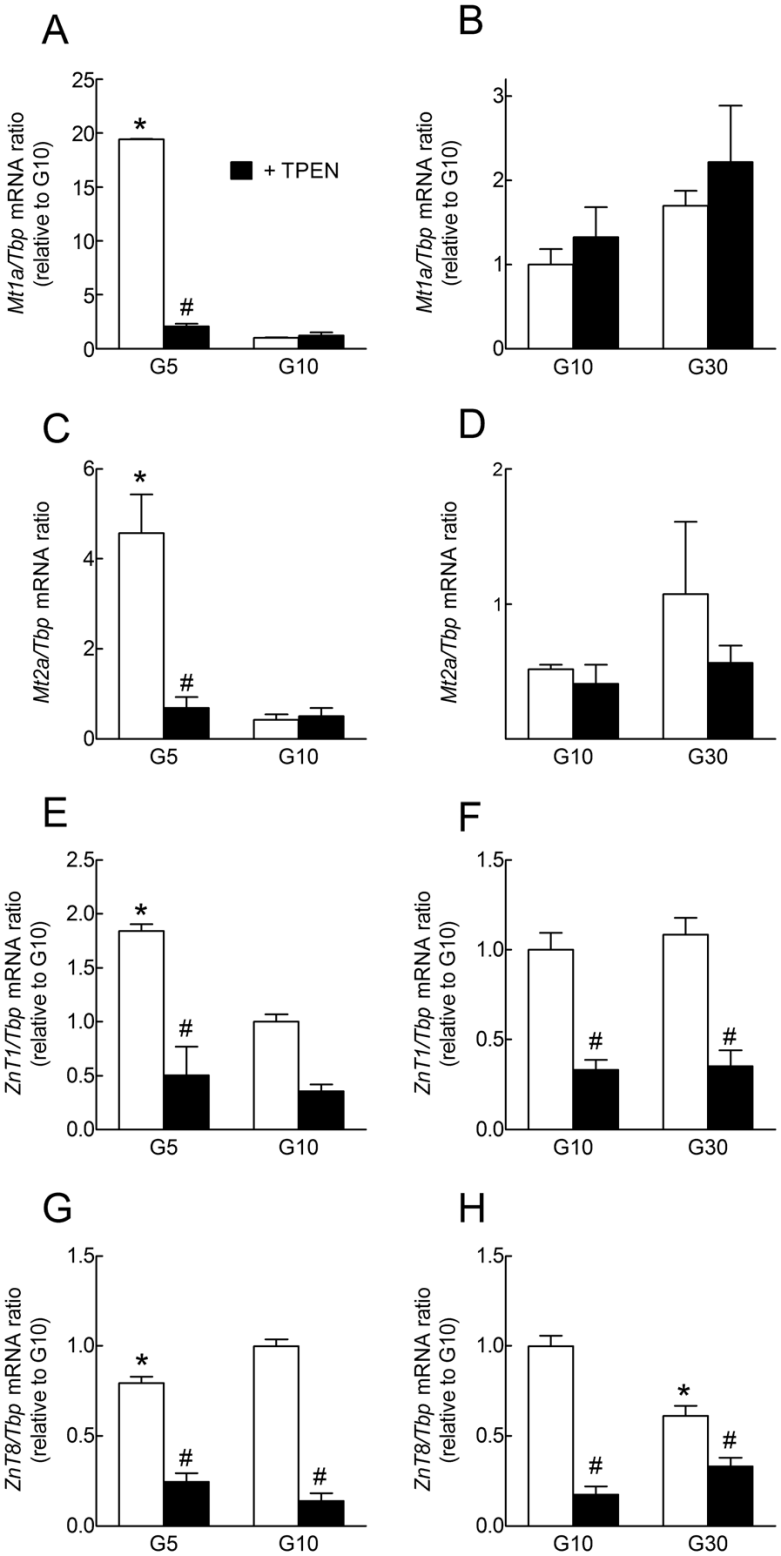
Effects of glucose and Zn^2+^ chelation by TPEN on *Mt1a, Mt2a, ZnT1 and ZnT8* mRNA expression in overnight cultured rat islets. Rat islets were precultured for 1 week in serum-free RPMI medium containing G10 and 5 g/l BSA. They were then cultured for 18 h in the presence of 5, 10 or 30 mmol/l glucose (G5, G10 or G30) with DMSO (open bars) or with 6.25 µmol/l TPEN (dissolved in DMSO) (closed bars). *Mt1a*, *Mt2a, ZnT1* and *ZnT8* to *Tbp* mRNA ratios were measured by real-time PCR. Except for *Mt2a*, the *Gene* to *Tbp* mRNA ratios were expressed relative to the ratio measured in islets cultured in G10 without TPEN. C, the mean Ct for *Tbp* was 27.5 in G5, 29.3 in G5+TPEN, 28 in G10 and 28.9 in G10+TPEN. D, the mean Ct for *Tbp* was 27.9 in G10, 28.8 in G10+TPEN, 27.8 in G30 and 29.2 in G30+TPEN. Results are means ± SEM for 3 experiments. *, *P*<0.05 for the effect of G5 *vs.* G10 and #, *P*<0.05 for the effect of TPEN by two-way ANOVA followed by a test of Bonferroni.

Metallothioneins are small proteins of ∼60 AA of which ∼20 are cysteine residues. Their gene expression is mainly regulated by metal transcription factors (MTF) that are activated by heavy metals and by a rise in free intracellular Zn^2+^ concentration ([Zn^2+^]_i_) [12]. Metallothioneins play an essential role in Zn^2+^ homeostasis by having the capacity to bind up to seven Zn^2+^ ions [13] and by controlling Zn^2+^ distribution to various Zn^2+^-binding proteins [14]. These functions are clearly regulated by changes in cellular redox state. Thus, while Zn^2+^ is preferentially bound to metallothioneins under reductive conditions, oxidation of their cysteine residues releases Zn^2+^ that can activate MTF, thereby increasing metallothionein expression and restoring cell Zn^2+^ buffering capacity [15]. In addition, the increase in [Zn^2+^]_i_ has also been proposed to exert antioxidant and antiapoptotic effects through various mechanisms, e.g. caspase inhibition, xanthine oxidase inhibition, increased cytosolic superoxide dismutase activity and metallothionein overexpression [16].

Zn^2+^ is an important cofactor for insulin biosynthesis and crystallization [17] and is co-secreted with insulin. A non-synonymous polymorphism in the *Slc30a8* gene encoding the insulin granule Zn^2+^ transporter Slc30a8 (ZnT8) has been associated with the risk of developing type 2 diabetes [18]. Interestingly, type 2 diabetic patients display a marked decrease in total plasma Zn^2+^ concentration together with hyperzincuria, and Zn^2+^ supplementation has been shown to ameliorate glycemic control in both type 1 and type 2 diabetes (reviewed in [19], [20]. A high-Zn^2+^ diet also improved blood glucose levels after islets transplantation in diabetic rats [21]. However, the mechanisms underlying these beneficial effects of Zn^2+^ have been poorly elucidated.

**Figure 2:**
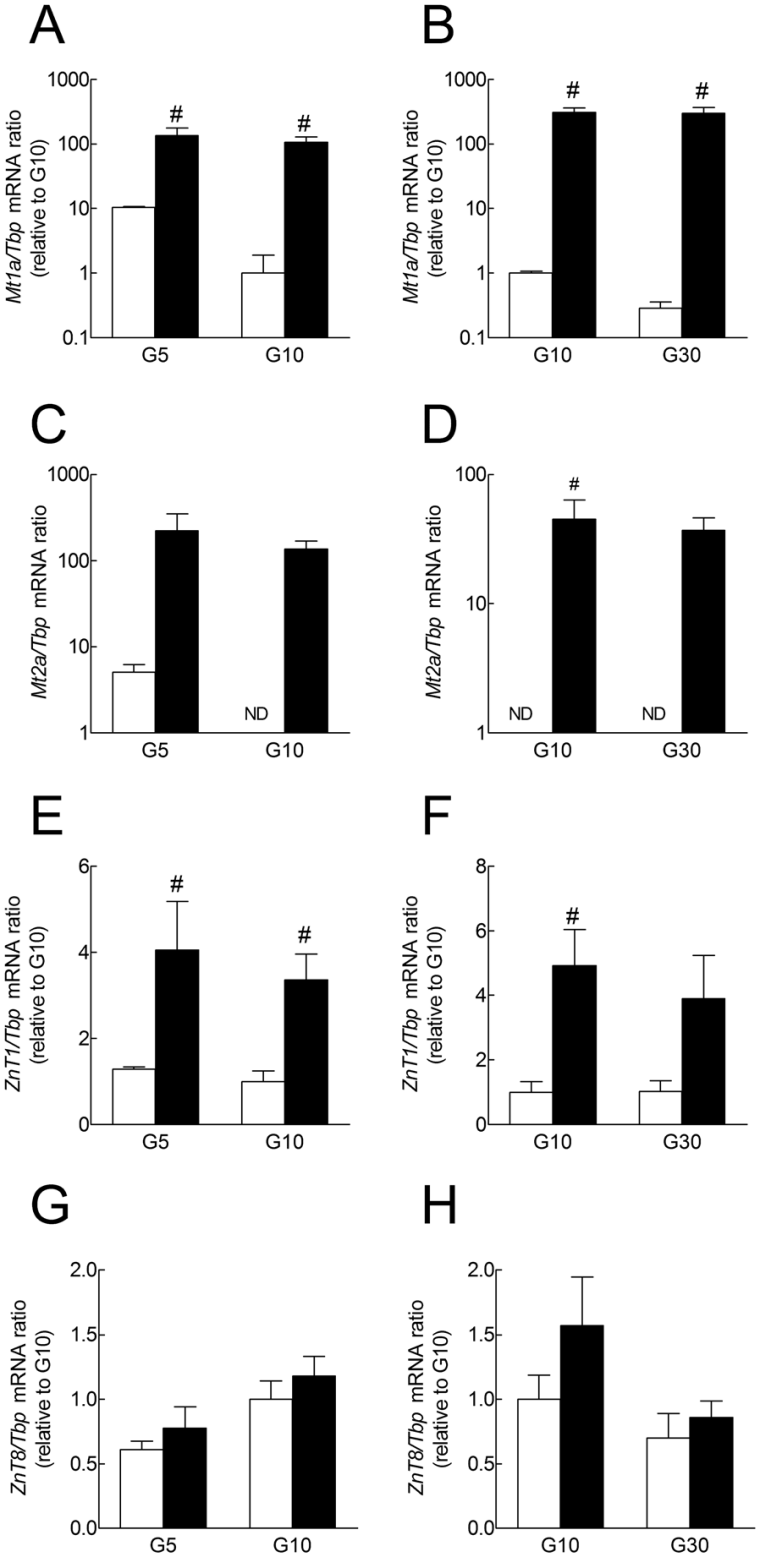
Long term effects of glucose and ZnCl_2_ on *Mt1a*, *Mt2a, ZnT1 and ZnT8* mRNA expression in cultured rat islets. After preculture, rat islets were cultured for 1 week in the presence of G5, G10 or G30 alone (open bars) or in the presence of 100 µmol/l ZnCl_2_ (closed bars). *Gene* to *Tbp* mRNA ratios were measured by real-time PCR and expressed relative to the ratio measured in islets cultured in G10 without ZnCl_2_, except for *Mt2a* to *Tbp* mRNA ratios that are shown without normalization. ND: Not Detected (unspecific products with lower Tm were amplified at Ct>30, i.e. at *Mt2a/Tbp* mRNA ratio <0.25). C, the mean Ct for *Tbp* were 27 in G5, 27.5 in G5+ZnCl_2_, 27.3 in G10 and 27.3 in G10+ZnCl_2_. D, the mean Ct for *Tbp* were 28.1 in G10, 28 in G10+ZnCl_2_, 27.8 in G30 and 28.1 in G30+ZnCl_2_. Please note the logarithmic Y-scale in A-D. Results are means ± SEM for 3 or 4 experiments. #, *P*<0.05 for the effect of ZnCl_2_ by two-way ANOVA followed by a test of Bonferroni. C,D, for statistical analysis of differences between groups, *Mt2a/Tbp* mRNA ratio was set to 0.25 when not detected.

We therefore tested the effect of ZnCl_2_, a potent inducer of metallothionein expression, on islet cell apoptosis and the alterations of glucose-stimulated insulin secretion (GSIS) after prolonged culture of rat islets in low and high *vs.* intermediate glucose concentrations. Using the mitochondria-targeted redox-sensitive ratiometric fluorescent probe mt-roGFP1, we also tested the effect of culture in the presence of low, intermediate and high glucose concentrations with or without ZnCl_2_ on β-cell mitochondrial thiol oxidation state.

## Materials and Methods

### Materials

Dithiothreitol (DTT) and N,N,N′N′-tetrakis(-)[2-pyridylmethyl]-ethylenediamine (TPEN) were purchased from Sigma (St-Louis, MO, USA). Hydrogen peroxide (H_2_O_2_) and ZnCl_2_ were obtained from Acros Organics (Thermo Fisher Scientific, New Jersey, USA). Other reagents of analytical grade were purchased from Merck (Darmstadt, Germany).

**Figure 3:**
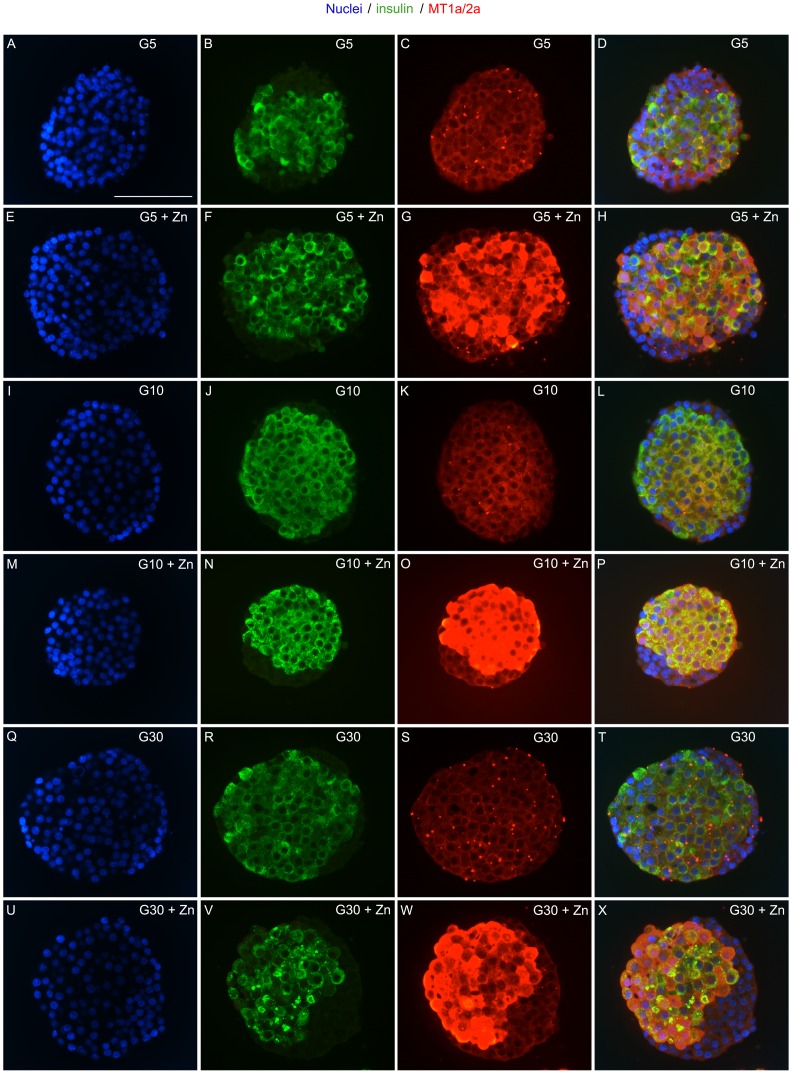
Long term effects of glucose and ZnCl_2_ on MT1a/2a protein levels in cultured rat islets. After preculture, rat islets were cultured for 1 week in the presence of G5, G10 or G30 alone or with 100 µmol/l ZnCl_2_. Islets were then fixed in paraformaldehyde solution (4%) and embedded in paraffin. Nuclei (DAPI), insulin and MT1a/2a were detected by immunohistochemistry in 5 µm-thick islets sections. Bar scale = 100 µm. A–D: islets cultured in G5; E–H : islets cultured in G5+100 µM ZnCl_2_ (G5+ Zn); I-L: islets cultured in G10; M-P: islets cultured in G10+100 µM ZnCl_2_ (G10+ Zn); Q–T: islets cultured in G30; U–X: islets cultured in G30+100 µM ZnCl_2_ (G30+ Zn). A,E,I,M,Q,U: DAPI staining; B,F,J,N,R,V: insulin staining; C,G,K,O,S,W: MT1a/2a staining; D,H,L,P,T,X: merge. Results are representative for 2 to 3 experiments.

### Islet Isolation and Culture

Pancreatic islets were isolated from ∼200 g male Wistar rats as described [4]. Except for experiments on cell clusters, the islets were precultured for one week at 37°C and 5% CO_2_ in serum-free RPMI 1640 (Invitrogen, Carlsbad, CA, USA) containing 5 g/l BSA and 10 mmol/l glucose. They were then cultured for up to 1 week in the same medium containing 5, 10 or 30 mmol/l glucose (G5, G10, or G30) and various test substances, and processed for further analysis. Experimental procedures were approved by the local ethics committee for animal experimentation.

### Real-time PCR

Islet gene mRNA levels were measured as described [22]. Briefly, islet total RNA was extracted using Tripure (Roche Diagnostics GmbH, Mannheim, Germany) and reversed transcribed into cDNA using 50 ng of randoms hexamers and 200 units of the enzyme RevertAid™ H Minus M-MuLV Reverse Transcriptase (Fermentas GmbH, St.Leon-Rot, Germany). Real-time PCR was performed with an iCycler iQ Real-Time PCR Detection System (Bio-Rad, Hercules,CA). Primers sequences and reactions conditions are shown in Table S1. Islet gene mRNA to TATA-box binding protein (*Tbp*) mRNA or cyclophilin mRNA ratios (2^−ΔCt^) were expressed relative to the ratio in islets cultured in G10.

**Figure 4:**
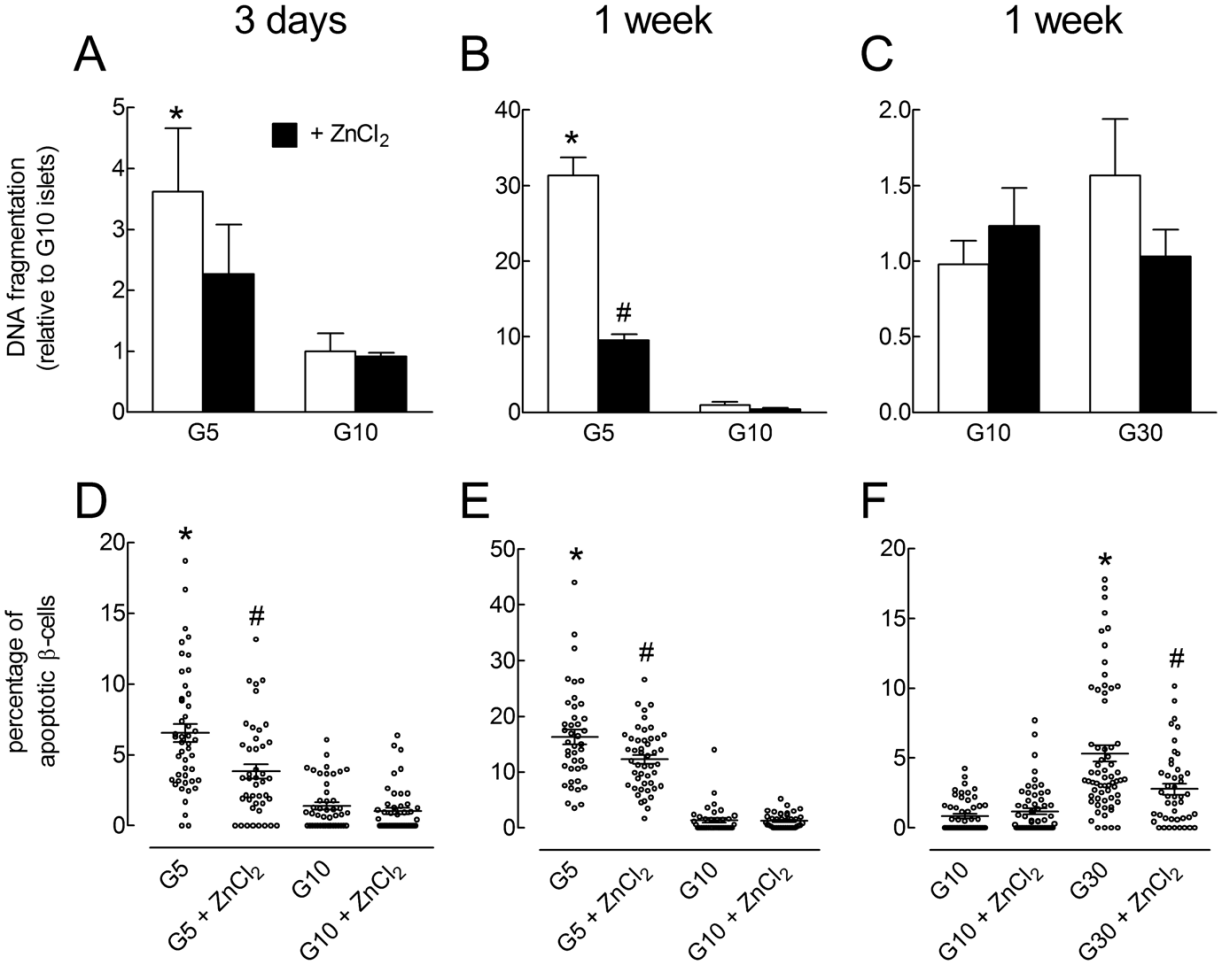
Effects of glucose and ZnCl_2_ on the stimulation of rat islet cell apoptosis by prolonged culture in low and high glucose concentrations. Rat islets were cultured for 3 days (A, D) or 1 week (B,C,E,F) in the presence of G5, G10 or G30±100 µmol/l ZnCl_2_ as indicated. A, B, C, DNA fragmentation was measured by an ELISA kit and expressed relative to the value measured in islets cultured in G10. D, E, F, the percentage of apoptotic β-cells was measured by TUNEL. Results are means ± SEM for 3–5 experiments. *, *P*<0.05 for the effect of G5 or G30 *vs.* G10 and #, *P*<0.05 for the effect of ZnCl_2_ by two-way ANOVA followed by a test of Bonferroni (A,B,C) or by one-way ANOVA followed by a test of Newman-Keuls (D,E,F).

### Immunodetection of MT1a/2a

Islets were fixed in 4% paraformaldehyde and embedded in paraffin. Five µm-thick sections were incubated overnight with a mouse anti-MT1a/2a antibody (Abcam, Cambridge, UK) diluted 1∶100, washed in Tris-buffered saline and incubated for 1 h with Alexa Red 594-conjugated goat anti-mouse IgG (Invitrogen, CA, USA) diluted 1∶200. On the second day, washed sections were incubated overnight with guinea-pig anti-insulin antibody (Invitrogen) diluted 1∶2000. On the third day, sections were washed and incubated for 1 h with Alexa Green 488-conjugated goat anti-guinea-pig antibody diluted 1∶200. Sections were then mounted with Vectashield-mounting medium containing 4′,6-diamidino-2-phenylindole (DAPI) (Vector Laboratories, Burlingame, CA) and visualized on a fluorescence microscope (FluoArc mounted on an Axioskop 40 microscope coupled to an HBO 100 camera; Carl Zeiss, Oberkochen, Germany) under standardized conditions (excitation/emission wavelengths: insulin: 475/540 nm; DAPI: 350/460 nm; MT1a/2a: 590/617 nm).

**Figure 5:**
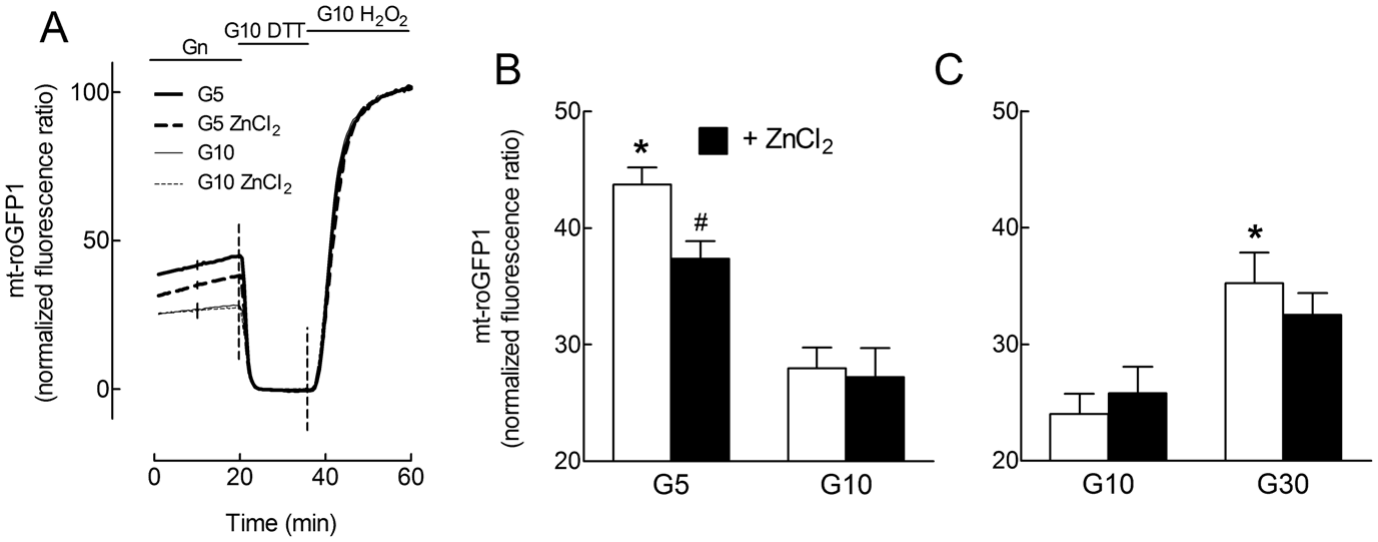
Effects of glucose and ZnCl_2_ on mitochondrial thiol/disulfide equilibrium in cultured rat islet cell clusters. Rat islet cell clusters infected with an adenovirus coding mt-roGFP1 were cultured overnight in the presence of G5, G10 or G30±50 µmol/l ZnCl_2_ as indicated. The ratio of mt-roGFP1 fluorescence intensities (exc 405/480 nm) was measured during 20 min of perfusion in a medium containing the same glucose concentration and expressed as a percentage of the difference between the mean ratio measured from 4 to 8 min after addition of 10 mmol/l DTT (set at 0%) and that measured from 14 to 18 min after addition of 1 mmol/l H_2_O_2_ (set at 100%) (B,C). Results are means ± SEM for 3 experiments (9–19 clusters). B,C, *, *P*<0.05 for the effect of G5 or G30 *vs.* G10 and #, *P*<0.05 for the effect of ZnCl_2_ by two-way ANOVA followed by a test of Bonferroni.

**Figure 6:**
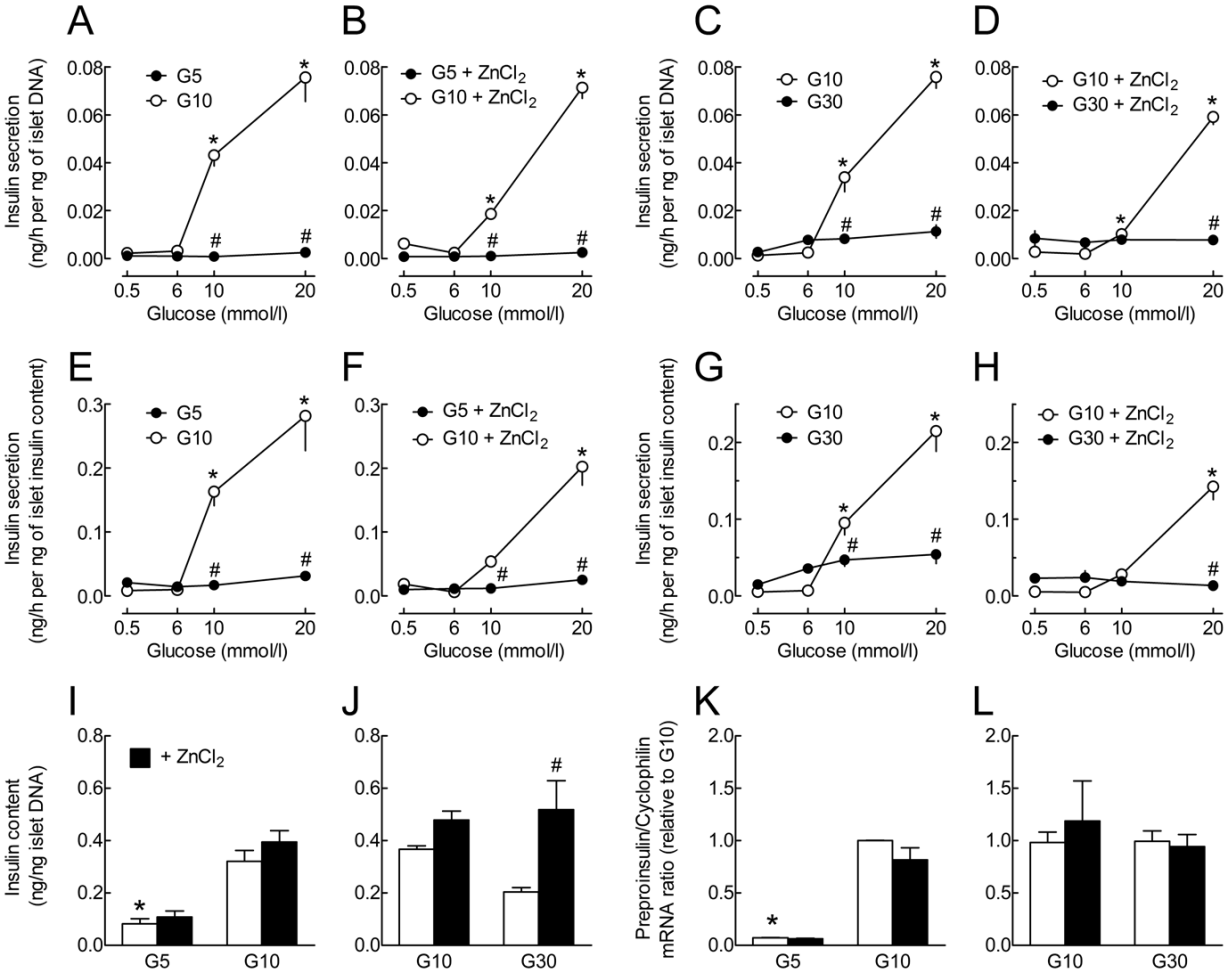
Effects of glucose and ZnCl_2_ during culture on subsequent glucose-stimulated insulin secretion and on preproinsulin mRNA expression. Islets were cultured for 3 days in the presence of G5 or G10 alone or with 100 µmol/l ZnCl_2_ (A,B,E,F,I), or for 1 week in the presence of G10 or G30 alone or with 100 µmol/l ZnCl_2_ (C,D,G,H,J). Batches of 5 islets were then pre-incubated for 45 min in G0.5 in the absence of ZnCl_2_ and next incubated for 1 h in the presence of G0.5, G6, G10 or G20. The islet insulin (I,J) and DNA content were measured at the end of the incubation. Insulin secretion was normalized for differences in islet DNA content (A–D) or for differences in islet insulin content (E–H). *, *P*<0.05 for the acute effect of glucose *vs.* G0.5 and ^#^, *P*<0.05 for the effect of culture in G5 or G30 *vs.* G10 by two-way ANOVA followed by a test of Bonferroni. I, J, *P<0.05 for the effect of culture in G5 and G30 *vs.* G10 and ^#^, *P*<0.05 for the effect of ZnCl_2_ during culture by two-way ANOVA followed by a test of Bonferroni. K–L, after preculture, rat islets were cultured for 1 week in G5, G10 or G30 alone (open bars) or in the presence of 100 µmol/l ZnCl_2_ (closed bars). Preproinsulin to cyclophilin mRNA ratios were measured by real-time PCR and expressed relative to the ratio measured in islets cultured in G10. K, the mean Ct for *cyclophilin* were 23.7 in G5, 24 in G5+ZnCl_2_, 24.1 in G10 and 24.4 in G10+ZnCl_2_. L, the mean Ct for *cyclophilin* were 23.7 in G10, 23.9 in G10+ZnCl_2_, 23.8 in G30 and 24 in G30+ZnCl_2_. Results are means ± SEM for 3 or 4 experiments. *, *P*<0.05 for the effect of culture in G5 *vs.* G10 by two-way ANOVA followed by a test of Bonferroni.

### Islet Cell Apoptosis

Cytoplasmic histone-associated DNA fragments were measured on batches of 50 to 60 islets using the Cell Death Detection ELISA^PLUS^ kit (Roche Diagnostics) as described [22]. The percentage of apoptotic β-cells (TUNEL-positive and insulin-positive cells) was determined on 5 µm-thick islet sections using the *In Situ* Cell Death Detection Kit - POD (Roche Diagnostics) following the manufacturer’s instructions. Sections were then incubated overnight with a guinea-pig anti-insulin antibody (1/1000) (Invitrogen, Eugene, Oregon, USA) and incubated for 1 h with an AlexaFluor 594-conjugated anti-guinea-pig antibody (1/1000) (Invitrogen, Camarillo, CA93012, USA). Islet cell nuclei were stained with DAPI. The percentage of apoptotic β-cells was determined by manually counting TUNEL, DAPI and insulin-positive nuclei on digital images obtained by fluorescence microscopy (FluoArc mounted on an Axioskop 40 coupled to a HBO 100 camera; Carl Zeiss, Oberkochen, Germany) under standardized conditions (excitation/emission wavelengths: fluorescein: 475/540 nm; DAPI: 350/460 nm; insulin: 590/617 nm).

### Adenovirus

Adenovirus encoding mt-roGFP1 under the control of the CMV promoter were generated and amplified using the pAdEasy system (Stratagene, La Jolla, CA), as previously described [11]. After purification on a gradient of CsCl, the infectious titre of viral stocks was determined with the Adeno-X™ Rapid Titer kit (Clontech, Mountain View, CA, USA).

### Mitochondrial Oxidative Stress Measurement

Mt-roGFP1, which measures the thiol/disulfide equilibrium in the mitochondrial matrix, was used as an indicator of mitochondrial redox status [23], [24]. After isolation, rat islets were dispersed in clusters using trypsin and gentle pipetting in a Ca^2+^-free medium. Cells clusters were plated on glass coverslips and cultured overnight in RPMI 1640 medium containing G10 and 10% Fetal Bovine Serum (FBS). Cells were infected for 48 h with adenovirus coding mt-roGFP1 (multiplicity of infection ∼25 to 50) and the medium was changed for the last 18 to 24 h with a medium containing G5 or G10 and FBS with or without 50 µM ZnCl_2_. Mt-roGFP1 fluorescence (excitation : 405 and 480 nm, emission : 535 nm, 40X objective) was measured every 30 s in cell clusters perifused with a bicarbonate-buffered Krebs solution containing (mmol/l) NaCl (120), KCl (4.8), CaCl_2_ (2.5), MgCl_2_ (1.2), NaHCO_3_ (24), 1 g/l BSA (fraction V, Roche, Basel, Switzerland) and continuously gassed with O_2_/CO_2_ (94/6) to maintain pH ∼7.4. For the first 20 min, cells were perifused with the same glucose concentration as during the last period of culture but without ZnCl_2_. They were then perifused with 10 mmol/l DTT for 15 min to maximally reduce the probe, followed by 1 mmol/l H_2_O_2_ for 25 min to maximally oxidize mt-roGFP1. The ratio of fluorescence intensities (exc 405/480) were computed and expressed as a percentage of the difference between the mean ratio measured from 4 to 8 min after addition of DTT (set at 0%) and that measured from 14 to 18 min after addition of H_2_O_2_ (set at 100%).

### Insulin Secretion

After culture, batches of 5 islets were incubated for 45 min in a bicarbonate-buffered Krebs solution containing 0.5 mmol/l glucose. Islets were then incubated for 1 h in the presence of various glucose concentrations. Insulin concentration in the medium was measured by RIA using rat insulin as a standard [25], and normalized for variations in islet DNA content measured by fluorimetry using SYBR Green I [26].

### Statistical Analysis

Results are means ± SEM for the indicated number of experiments. Statistical significance of differences between groups was assessed by one-way ANOVA followed by a test of Newman-Keuls or by two-way ANOVA followed by a test of Bonferroni, as indicated in the legends. Differences were considered significant if *P*<0.05.

## Results

### Effects of Glucose and Zn^2+^ Chelation on Mt1a, Mt2a, ZnT1 and Znt8 mRNA Expression in Overnight Cultured Rat Islets

After overnight culture of whole rat islets in the presence of 5, 10 or 30 mmol/l glucose (G5, G10 or G30), islet gene mRNA levels for the typical MTF-target genes *Mt1a* and *Mt2a* were minimal in G10, largely increased in G5, and slightly but not significantly increased in G30 (Fig. 1A–D). In comparison, the mRNA levels of the other MTF-target gene *Slc30a1* (*Znt1)* only increased ∼2-fold in G5 *vs.* G10 and were not affected by G30 (Fig. 1EF). In contrast, *Slc30a8* (*ZnT8*) mRNA expression decreased after culture in either G5 or G30 *vs.* G10 (Fig. 1GH). Under these conditions, the membrane-permeable Zn^2+^ chelator TPEN almost fully inhibited the stimulation of *Mt1a*, *Mt2a* mRNA expression by culture in G5, suggesting that a rise in [Zn^2+^]_i_ is involved in this effect of low glucose. In contrast, TPEN failed to affect *Mt1a* and *Mt2a* mRNA levels in G10 and G30 but significantly reduced *Znt1* and *ZnT8* mRNA levels under all conditions. These effects of TPEN were, however, accompanied by a ∼2 to 4-fold reduction in the mRNA levels of the housekeeping genes *Tbp* and cyclophilin under all culture conditions, and by a clear increase in islet cell apoptosis (not shown).

### Long-term Effects of Glucose and ZnCl_2_ on Mt1a, Mt2a, Znt1 and ZnT8 mRNA Expression in Cultured Rat Islets

After one week of culture in G5, *Mt1a* mRNA expression increased ∼10 fold in comparison with islets cultured in G10 (Fig. 2A). However, contrasting with data obtained after 18 h of culture, *Mt1a* mRNA expression did not increase and even tended to decrease after one week of culture in G30 *vs.* G10 (Fig. 2B). Similar results were obtained for *Mt2a* while *Znt1* and *ZnT8* mRNA levels were not affected by one week of culture at different glucose concentrations (Fig. 2C–H). Under these conditions, addition of 100 µmol/l ZnCl_2_ to the medium increased *Mt1a, Mt2a* and *Znt1* but not *ZnT8* mRNA expression at all glucose concentrations, *Mt1a* and *Mt2a* mRNA reaching levels at least a 100-fold higher than in islets cultured in G10 (Fig. 2). In comparison, addition of 10 or 30 µmol/l ZnCl_2_ to G5 only slightly increased *Mt1a* mRNA expression 1.9±0.4 and 2.2±0.6 times respectively (20±4 and 24±7 times the level in G10-cultured islets, n = 3). We therefore used ZnCl_2_ at the concentration of 100 µmol/l in all subsequent experiments carried out with whole islets.

### Long-term Effects of Glucose and ZnCl_2_ on MT1a/2a Protein Levels in Cultured Rat Islets

As shown in figure 3, MT1a/2a protein levels were not detectably affected by one week of culture in G5 or G30 *vs.* G10. However, addition of 100 µM ZnCl_2_ strongly increased MT1a/2a protein levels specifically in β-cells at all glucose concentrations.

### Effects of ZnCl_2_ on Rat Islet Cell Apoptosis Induced by Prolonged Culture in Low and High Glucose

We next tested the effect of ZnCl_2_ on islet cell apoptosis induced by prolonged culture in extreme glucose concentrations. As shown in figure 4AD, three days of culture in G5 instead of G10 induced a ∼3.5-fold increase in cytoplasmic histone-associated DNA fragments and a ∼4.5-fold increase in the percentage of TUNEL-positive β-cell nuclei. Addition of ZnCl_2_ during culture significantly reduced islet cell DNA fragmentation by ∼38% and the percentage of apoptotic β-cells by ∼68%. After one week of culture in G5, DNA fragmentation increased ∼30-fold while the percentage of apoptotic β-cells increased ∼12 fold (Fig. 4BE), and addition of ZnCl_2_ to the medium significantly reduced these effects by 27% and 70% respectively. After one week of culture in G30, islet cytoplasmic DNA fragments only tended to increase ∼1.5 fold, but the percentage of apoptotic β-cells was ∼6 times higher than in islets cultured in G10 (Fig. 4CF). Under these conditions, ZnCl_2_ reduced the stimulation of DNA fragmentation by ∼90% and the increase in the proportion of TUNEL-positive β-cells by 65%. These results indicate that 100 µmol/l ZnCl_2_ exerts a protective effect against rat β-cell apoptosis induced by chronic exposure to extreme glucose concentrations.

### Effects of Glucose and ZnCl_2_ on Mitochondrial Oxidative Stress in Rat Islet Cell Clusters

As ZnCl_2_ has been proposed to improve cell resistance to oxidative stress, we next tested its effect on early mitochondrial oxidative stress induced by extreme glucose concentrations in rat islet cell clusters expressing mt-roGFP1. In these preparations, the concentration of ZnCl_2_ had to be reduced to 50 µmol/l to avoid a strong increase in mt-roGFP1 oxidation in G10-cultured clusters (data not shown). As shown in figure 5BC, overnight culture in the presence of G5 or G30 instead of G10 significantly increased mt-roGFP1 oxidation in rat islet cell clusters, reflecting an increase in thiol (e.g. glutathione) oxidation in the mitochondrial matrix. Addition of ZnCl_2_ to the culture medium diminished by ∼40% the level of mt-roGFP1 oxidation in G5, and tended to reduce by ∼23% that in G30. These results suggest that ZnCl_2_ exerts some antioxidant effect in rat islet cell clusters exposed to low and high *vs.* intermediate glucose concentrations.

### Effects of ZnCl_2_ on the Alterations of Glucose-induced Insulin Secretion after Prolonged Culture in Low and High Glucose

We finally tested the effects of ZnCl_2_ during culture on islet insulin content and acute GSIS. As shown in figure 6A,B,E,F,I, three days of culture in the presence of G5 decreased the islet insulin content by ∼75%, decreased the absolute rate of insulin secretion in G0.5 and G6, and profoundly inhibited its stimulation by G10 and G20. Addition of ZnCl_2_ to the culture medium did not affect the islet insulin content or GSIS of islets cultured in G5, but significantly reduced the acute insulin secretory response to G10 of islets cultured in G10. In comparison with culture in G5, one week culture in G30 only reduced the islet insulin content by ∼30% and differently affected the GSIS, with a slight increase in the rate of insulin secretion in the presence of G6, and a reduced stimulation of insulin secretion in response to G10 and G20 (Fig. 6C,D,G,H,J). Although ZnCl_2_ prevented the decrease in islet insulin content during culture in G30, it did not improve their GSIS. The increase in insulin content was not due to an increase in preproinsulin mRNA levels (Figure 6K,L). Thus, despite the beneficial effect of ZnCl_2_ on β-cell survival, ZnCl_2_ did not protect against the alterations of GSIS induced by culture in extreme glucose concentrations.

## Discussion

We have previously shown that prolonged culture of rat islets in the presence of low or high *vs.* intermediate glucose concentrations rapidly induces the expression of oxidative stress-response genes such as *Mt1a* and *Hmox1*, followed by later increase in islet cell apoptosis and marked reduction of GSIS [4]. We now provide further evidence that these changes are associated with early parallel changes in mitochondrial oxidative stress, and demonstrate that ZnCl_2_, which potently induces *Mt1a* and *Mt2a* expression, exerts a protective effect on mitochondrial thiol oxidation and subsequent β-cell apoptosis without improving GSIS.

In isolated rat islets cultured overnight in the presence of increasing glucose concentrations, the mRNA levels of the MTF-target genes *Mt1a* and *Mt2a* were minimal in G10, markedly increased in G5, and tended to increase in G30 *vs.* G10. These glucose effects were associated with parallel changes in mt-roGFP1 oxidation, a good indicator of thiol (mainly glutathione) oxidation in the mitochondrial matrix [11], [27], suggesting the presence of mitochondrial oxidative stress after 18h culture in either low or high *vs.* intermediate glucose concentrations. We have recently shown that mt-roGFP1 oxidation is acutely stimulated in rat islet cell clusters upon a reduction in glucose concentration from 10 to 2 mmol/l but that it is not increased upon glucose stimulation from 10 to 30 mmol/l glucose [11]. Together with the present study, these data indicate that the stimulation of oxidative stress induced by G30 is slower than that induced by G5, as is the case for the stimulation of β-cell apoptosis under these culture conditions [4]. However, in both G5 and G30, mt-roGFP1 oxidation occurred earlier than β-cell apoptosis, suggesting that the latter may result from mitochondrial oxidative stress. In that scenario, we postulate that the increase in *Mt1a* and *Mt2a* mRNA levels are sensitive indicators of this type of stress. Although we did not fully investigate the mechanism of *Mt1a* and *Mt2a* mRNA inductions, it is possible that oxidation of metallothioneins releases Mt-bound Zn^2+^, with consequent activation of metal transcription factor-1 (MTF-1) and increased expression of its target genes *Mt1a*, *Mt2a* and *Znt1* [28], [29]. In comparison, expression of the type 2 diabetes gene *Slc30a8* that encodes the β-cell specific granular zinc transporter *ZnT8* was not regulated in parallel with *Znt1* or *Mts*, in agreement with the observations that it is not induced by ZnSO_4_ [30] nor by ZnCl_2_ (the present study). Despite the large changes in *Mt1a* and *Mt2a* mRNA levels, MT1a/2a protein levels were not detectably increased by culture in low or high *vs.* intermediate glucose concentrations. This discordance between changes in Mt mRNA and protein levels could result from the low sensitivity of immunohistochemistry and, at least in low glucose, from a global decrease in protein translation in G5 *vs.* G10 [31].

It has recently been shown that the free cytosolic Zn^2+^ concentration ([Zn^2+^]_c_) in CD1 mouse islets decreases from ∼800 to ∼400 pmol/l after 24 h culture in 3 *vs.* 16.7 mmol/l glucose while *Mt1-2* mRNA levels increase and ZiP6-8 expression decreases under these conditions [32]. Although these results seem to argue against a role of a rise in [Zn^2+^]_c_ in the stimulation of *Mt* gene expression by culture in low glucose, our hypothesis is strongly supported by the observation that the membrane-permeable Zn^2+^ chelator TPEN fully suppressed the induction of MTF-target gene expression by G5. Thus, we interpret the late decrease in [Zn^2+^]_c_ measured by Bellomo et al. after 24 h culture in 3 *vs.* 16.7 mmol/l glucose as a possible consequence of the increase in metallothionein expression (although it was not detected by immunohistochemistry) and Zn^2+^-buffering capacity under these culture conditions. Alternatively, glucose-induced changes in [Zn^2+^] might be different in the cytosolic and nuclear compartments of islet cells [33], or between mouse and rat β-cells.

It has previously been shown that Zn^2+^ supplementation reduces early graft failure in diabetic rats transplanted with syngeneic islets [21], protects mice from diabetes induced by multiple low doses of streptozotocin [34] and ameliorates glucose tolerance in type 1 and type 2 diabetic patients [19], [35], but the underlying mechanisms are not clear [20]. Also *in vitro*, addition of ZnSO_4_ to the culture medium partially protected islet cells against the toxic effect of streptozotocin [36]. In the present study, addition of 50–100 µmol/l ZnCl_2_ to the culture medium significantly reduced mitochondrial thiol oxidation and β-cell apoptosis triggered by prolonged exposure to low or high *vs.* intermediate glucose concentrations. These effects were unlikely due to the negligible increase in chloride anions but rather resulted from the provision of Zn^2+^, a trace element surprisingly absent from standard RPMI medium. In another study in which medium contained 5 mg/ml BSA as in ours, ∼75% of Zn^2+^ was bound to BSA [37]. Thus, the antiapoptotic effects of Zn^2+^ on whole islets were observed at a concentration similar to that measured in rodent plasma [37] but lower than those reported to exert proapoptotic effects in β-cells or other cell types [38]–[41]. Differences in Zn^2+^ binding or the addition of Zn^2+^ through FBS may explain the need to reduce ZnCl_2_ concentration at 50 µmol/l to avoid apoptosis of islet cell clusters. Although it has been shown that addition of 90 µmol/l ZnSO_4_ does not increase the intracellular zinc content of INS-1E cells [41], a recent study using a new zinc-sensitive fluorescent protein in mouse islet cell clusters has recently demonstrated that addition of 50 µmol/l ZnCl_2_ approximately doubled their [Zn^2+^]_c_ while increasing *Mt1* gene expression [32]. It is therefore likely that ZnCl_2_ treatment induces a rise in [Zn^2+^]_c_ in rat as in mouse islets.

The beneficial effect of ZnCl_2_ on mitochondrial glutathione oxidation after culture in low (and to some extent high) *vs.* intermediate glucose concentrations may contribute to its antiapoptotic effect in β-cells. Although Zn^2+^ deficiency has been shown to increase oxidative stress in other cell types, few studies have demonstrated that Zn^2+^ decreases oxidative stress (reviewed in [42]). The mechanism could involve the increase in metallothionein expression, a group of proteins that play a role in Zn^2+^ distribution to the lipids and in protection of the proteins damaged by oxidative stress [15]. *Mts* are also known to be ROS scavengers with a capacity to capture hydroxyl radicals ∼50 times greater than that of glutathione [43]. The mechanism could also involve the role of Zn^2+^ in Cu/Zn superoxide dismutase (SOD1) activity, an enzyme which removes the superoxide radical, although it has been shown that Zn^2+^ deficiency does not decrease SOD1 activity [44], [45]. In addition, other pathways, such as the inhibition of caspase 3, 6 and 9 could play a role in the antiapoptotic effect of Zn^2+^ (reviewed in [42]).

Despite the significant improvement in β-cell survival, Zn^2+^ did not have any protective effect on the alterations of GSIS induced by prolonged culture in extreme glucose concentrations, except for a significant increase in the insulin content of high glucose-cultured islets. Actually, addition of ZnCl_2_ during culture even decreased subsequent insulin secretion in the presence of 10 mmol/l glucose, an effect that was more pronounced when insulin secretion was expressed relative to the islet insulin content. These effects, including the increase in insulin content after culture in 30 mmol/l glucose, could result from the inhibition of Ca^2+^ influx and GSIS by Zn^2+^ in a slowly reversible manner [37], [46], [47]. Alternatively, we cannot exclude that the inhibition of insulin secretion by ZnCl_2_ in G10 indirectly resulted from an inhibition of glucagon secretion by α-cells (reviewed in [48]), hence of [cAMP] in β-cells. However, a similar lack of improvement of GSIS was observed when islet cell apoptosis triggered by prolonged culture in 5 instead of 10 mmol/l glucose was inhibited by 50–70% with the SOD and catalase-mimetic manganese (III)tetrakis (4-benzoic acid)porphyrin (MnTBAP) [49]. Two hypotheses may explain these results. Either β-cell function is more sensitive than survival to the remaining level of oxidative stress present in the presence of Zn^2+^ or MnTBAP, or the loss of GSIS under these culture conditions is unrelated to mitochondrial oxidative stress. Thus, although an increase in β-cell mass with no alteration in secretory function may contribute to the beneficial effect of Zn^2+^ supplementation on glucose tolerance in diabetes, our results emphasize the importance of testing GSIS in addition to cell survival when testing potential treatments of stressed β-cells.

In conclusion, culture of rat pancreatic islets in either low or high *vs.* intermediate glucose concentrations triggers early mitochondrial oxidative stress with *Mt1a/2a* mRNA expression and late β-cell apoptosis with loss of GSIS. ZnCl_2_ reduces mitochondrial oxidative stress and rat β-cell apoptosis under these culture conditions without improving GSIS.

## Supporting Information

Table S1 **Sequences of oligonucleotide primers and PCR conditions.** The specificity of sense and anti-sense primers was checked by BLAST search. The thermal cycle profile consisted of a 3 min step at 95°C to release DNA polymerase activity followed by 40 cycles of amplification (15 sec denaturation step at 95°C, 45-60-90 sec annealing step at 60–62°C, and eventual 15–30 sec extension step at 80-82-84°C). Under these conditions, PCR efficiencies were ∼0.95 to 1.0. The melting temperature (T_m_) of the amplicons was systematically determined at the end of the PCR to check their specificity. Their size corresponded to that expected from published sequences, as determined by agarose gel electrophoresis. ^*^, Islet sample cDNA input in 25 µl reactions (ng total RNA equivalent).
(DOC)
