# Supplementary material for: Protective Antioxidant and Antiapoptotic Effects of ZnCl2 in Rat Pancreatic Islets Cultured in Low and High Glucose Concentrations
Source: PLoS One. 2012 Oct 3;7(10):e46831. doi: 10.1371/journal.pone.0046831 (PMC3463538; doi:10.1371/journal.pone.0046831)
Supplement: Table S1 — Sequences of oligonucleotide primers and PCR conditions. The specificity of sense and anti-sense primers was checked by BLAST search. The thermal cycle profile consisted of a 3 min step at 95°C to release DNA polymerase activity followed by 40 cycles of amplification (15 sec denaturation step at 95°C, 45-60-90 sec annealing step at 60–62°C, and eventual 15–30 sec extension step at 80-82-84°C). Under these conditions, PCR efficiencies were ∼0.95 to 1.0. The melting temperature (Tm) of the amplicons was systematically determined at the end of the PCR to check their specificity. Their size corresponded to that expected from published sequences, as determined by agarose gel electrophoresis. *, Islet sample cDNA input in 25 µl reactions (ng total RNA equivalent). (DOC) [file pone.0046831.s001.doc]

| Gene | Sense primer (5’-3’) | Antisense primer (5’-3’) | Input ^*^ | Annealing (°C, sec) | Extension (°C, sec) | Size (bp) | T_m_ (°C) |
| --- | --- | --- | --- | --- | --- | --- | --- |
| *Tbp* | ACCCTTCACCAATGACTCCTATG | TCAGCATTTCTGGCACGAAGT | 2 | 60-45 | 80-15 | 157 | 84 |
| *Mt1a* | GGACCCCAACTGCTCCCT | CGAGGCACCTTTGCAGACAC | 2 | 62-45 | 82-15 | 160 | 89.5 |
| *Mt2a* | CAGCGATCTCTCGTTGATCTCC | CTTGTCCGAAGCCTCTTTGC | 2 | 60-60 | - | 210 | 89.5 |
| *ZnT1* | TGGGCTCTGTGATCGTTGTT | GCACAGGGTTGGATCTAGGTA | 2 | 60-60 | - | 194 | 86.5 |
| *ZnT8* | CCCGACTACAAAATGGCTGATC | CAGCGACCAGATGTGTAGATTGT | 2 | 62-60 | - | 201 | 85.5 |
| Preproinsulin | TCTTCTACACACCCATGTCCC | GGTGCAGCACTGATCCAC | 0.5 | 60-90 | 84-30 | 148 | 91 |
| Cyclophilin | AACCCCACCGTGTTCTTC | TGCCTTCTTTCACCTTCC | 0.5 | 60-90 | 84-30 | 400 | 87.5 |
